# Prostaglandin D_2_ Induces Ca^2+^ Sensitization of Contraction without Affecting Cytosolic Ca^2+^ Level in Bronchial Smooth Muscle

**DOI:** 10.3390/ijms19103036

**Published:** 2018-10-05

**Authors:** Wataru Suto, Yusuke Ando, Takahiro Hirabayashi, Fumiko Takenoya, Seiji Shioda, Junzo Kamei, Hiroyasu Sakai, Yoshihiko Chiba

**Affiliations:** 1 Department of Physiology and Molecular Sciences, School of Pharmacy, Hoshi University, 2-4-41 Ebara, Shinagawa-ku, Tokyo 142-8501, Japan; d1502@hoshi.ac.jp (W.S.); kuki@hoshi.ac.jp (F.T.); 2 Global Research Center for Innovative Life Science, School of Pharmacy, Hoshi University, 2-4-41 Ebara, Shinagawa-ku, Tokyo 142-8501, Japan; p17andoh@hoshi.ac.jp (Y.A.); kamei@hoshi.ac.jp (J.K.); 3 Peptide Drug Innovation Global Research Center for Innovative Life Science, School of Pharmacy, Hoshi University, 2-4-41 Ebara, Shinagawa-ku, Tokyo 142-8501, Japan; t-hirabayashi@hoshi.ac.jp (T.H.); shioda@hoshi.ac.jp (S.S.); 4 Department of Biomolecular Pharmacology, School of Pharmacy, Hoshi University, 2-4-41 Ebara, Shinagawa-ku, Tokyo 142-8501, Japan; sakai@hoshi.ac.jp

**Keywords:** bronchial smooth muscle hyperresponsiveness, prostaglandin D_2_ (PGD_2_), DP_1_ receptor, Ca^2+^ sensitization, RhoA

## Abstract

Prostaglandin D_2_ (PGD_2_) is one of the key lipid mediators of allergic airway inflammation, including bronchial asthma. However, the role of PGD_2_ in the pathogenesis of asthma is not fully understood. In the present study, the effect of PGD_2_ on smooth muscle contractility of the airways was determined to elucidate its role in the development of airway hyperresponsiveness (AHR). In isolated bronchial smooth muscles (BSMs) of naive mice, application of PGD_2_ (10^−9^–10^−5^ M) had no effect on the baseline tension. However, when the tissues were precontracted partially with 30 mM K^+^ (in the presence of 10^−6^ M atropine), PGD_2_ markedly augmented the contraction induced by the high K^+^ depolarization. The PGD_2_-induced augmentation of contraction was significantly inhibited both by 10^−6^ M laropiprant (a selective DP_1_ antagonist) and 10^−7^ M Y-27632 (a Rho-kinase inhibitor), indicating that a DP_1_ receptor-mediated activation of Rho-kinase is involved in the PGD_2_-induced BSM hyperresponsiveness. Indeed, the GTP-RhoA pull-down assay revealed an increase in active form of RhoA in the PGD_2_-treated mouse BSMs. On the other hand, in the high K^+^-depolarized cultured human BSM cells, PGD_2_ caused no further increase in cytosolic Ca^2+^ concentration. These findings suggest that PGD_2_ causes RhoA/Rho-kinase-mediated Ca^2+^ sensitization of BSM contraction to augment its contractility. Increased PGD_2_ level in the airways might be a cause of the AHR in asthma.

## 1. Introduction

Augmented airway responsiveness to a wide variety of nonspecific stimuli, called airway hyperresponsiveness (AHR), is a common feature of allergic asthma. A cause of the AHR is hypercontraction of smooth muscle cells of the airways [1,2,3,4,5]. Rapid remission from airway limitation in asthma attack by inhalation of short-acting beta-stimulant also suggests an involvement of increased airway smooth muscle contraction in the airway obstruction. It is thus important for development of asthma therapy to understand the disease-associated alterations of the contractile signaling of airway smooth muscle cells.

Prostaglandin D_2_ (PGD_2_), one of the cyclooxygenase (COX) metabolites, is the major lipid mediator released from mast cells in allergic reaction, and has been suggested to be involved in the pathogenesis of bronchial asthma. An increase in PGD_2_ level in bronchoalveolar lavage (BAL) fluids was demonstrated in experimental asthma models [6,7]. In asthmatic subjects, allergen challenge to the airways caused an increase in PGD_2_ in BAL fluids [8,9]. It has been suggested that PGD_2_ mediates allergic inflammation, including the airway inflammation in asthma. In mice lacking receptors for PGD_2_ (DP_1_ receptors), both airway eosinophilia and upregulation of proinflammatory cytokines in BAL fluids induced by allergen challenge were diminished as compared to wild-type animals [10]. PGD_2_ also caused cytokine release via an activation of a PGD_2_ receptor, CRTH2 (also called as DP_2_ receptor), in Th2 lymphocytes [11].

On the other hand, the functional role of PGD_2_ on airway smooth muscle remains unclear. Application of PGD_2_ to the isolated smooth muscle strips caused contraction in guinea pig trachea [12] and dog bronchus [13]. In contrast, PGD_2_ elicited a relaxation in the murine tracheal smooth muscle precontracted with carbachol [10]. In the present study, to elucidate its role in the development of AHR in asthma, the effect of PGD_2_ on smooth muscle contractility was determined using bronchial rings isolated from mice.

## 2. Results

### 2.1. Effects of Prostaglandin D_2_ (PGD_2_) on Bronchial Smooth Muscle (BSM) Function

**Figure 1:**
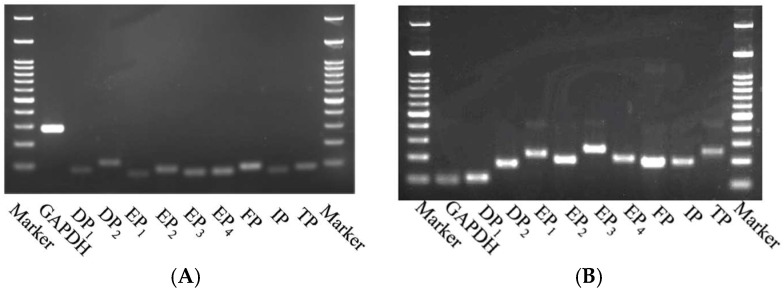
Expression of various prostanoid receptors in cultured human bronchial smooth muscle (BSM) cells (**A**) and murine BSMs (**B**) determined by RT-PCR analyses. Marker: M.W. markers (100 bp ladder), GAPDH: glyceraldehyde-3-phosphate dehydrogenase, DP_1_: prostaglandin D_2_ (PGD_2_) receptor 1, DP_2_: PGD_2_ receptor 2, EP_1_: PGE_2_ receptor 1, EP_2_: PGE_2_ receptor 2, EP_3_: PGE_2_ receptor 3, EP_4_: PGE_2_ receptor 4, FP: PGF_2_ receptor, IP: PGI_2_ receptor, and TP: thromboxane A_2_ receptor. The primer sets used are shown in Materials and Methods section.

**Figure 2:**
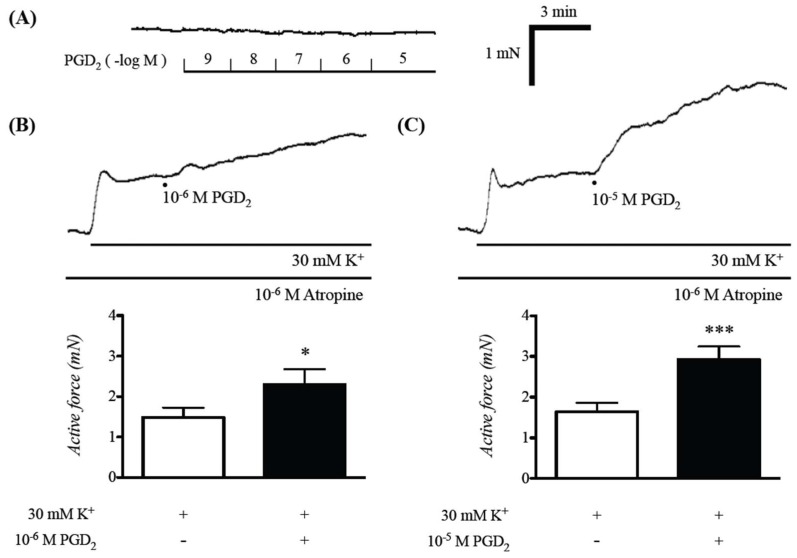
Effects of prostaglandin D_2_ (PGD_2_) on the contraction induced by 30 mM K^+^ depolarization in bronchial smooth muscles (BSMs) isolated from mice. PGD_2_ (10^−9^–10^−5^ M) had no effect on basal tone (**A**). After the stable contraction induced by K^+^ depolarization was observed, 10^−6^ (**B**) or 10^−5^ M (**C**) PGD_2_ was applied. Representative traces of changes in the active force are shown in respective upper panels, and the data are summarized in the lower panels. Results are presented as mean ± SEM from 5 animals, respectively. * *p* < 0.05 and *** *p* < 0.001 versus without PGD_2_ by paired Student’s *t*-test.

The RT-PCR analyses revealed that both DP_1_ and DP_2_ receptors were expressed both in hBSMCs and murine BSMs (Figure 1), indicating that PGD_2_ could directly act on BSM cells. To determine the role of PGD_2_ on the BSM function, its effect on the isometric tension of smooth muscles was examined in BSM tissues isolated from naive control mice. Application of PGD_2_ (10^−9^–10^−5^ M) had no effect on basal tone of the BSM tissues (Figure 2A). However, when the BSMs were precontracted with 30 mM K^+^, application of PGD_2_ caused an enhancement of the contraction induced by high K^+^ depolarization, in a PGD_2_ concentration-dependent manner (10^−6^ and 10^−5^ M: Figure 2B,C).

**Figure 3:**
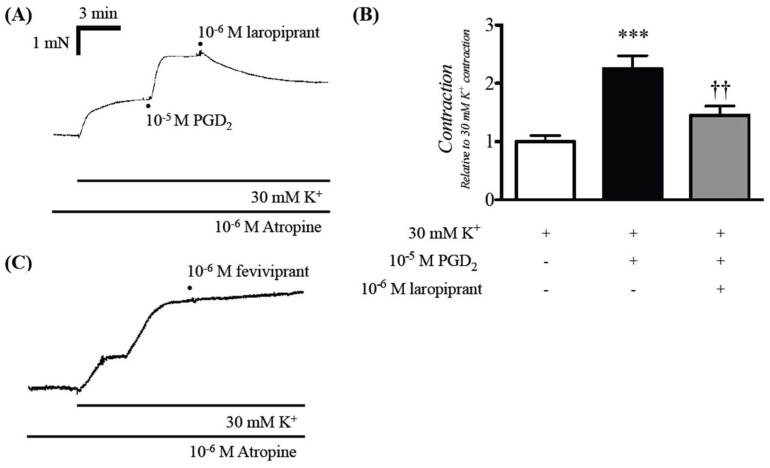
Effect of laropiprant (a selective DP_1_ receptor antagonist) on the augmented contraction induced by prostaglandin D_2_ (PGD_2_) in bronchial smooth muscles (BSMs) isolated from mice. After the BSM contraction induced by PGD_2_ reached to plateau, 10^−6^ M laropiprant was applied. Representative traces of changes in the active force are shown in (**A**), and the data are summarized in (**B**). Results are presented as mean ± SEM from 5 animals. *** *p* < 0.001 versus 30 mM K^+^ only group and ^††^ *p* < 0.01 versus 30 mM K^+^ + 10^−5^ M PGD_2_ group by one-way ANOVA with post hoc Bonferroni’s multiple comparison. Note that fevipiprant (10^−6^ M, a selective DP_2_ receptor antagonist) had no effect on the PGD_2_-induced augmentation of contraction (**C**).

PGD_2_ has been known to act on G protein-coupled receptors (GPCRs), mainly the PGD_2_ receptor 1 (DP_1_) and 2 (DP_2_). To elucidate receptor(s) responsible for the enhanced contraction induced by PGD_2_, effects of laropiprant (a selective DP_1_ receptor antagonist [14]) and fevipiprant (a selective DP_2_ receptor antagonist [15]) on the PGD_2_-induced augmentation of contraction were tested. As a result, the enhanced contraction induced by PGD_2_ was inhibited by laropiprant (10^−6^ M: Figure 3A,B), whereas fevipiprant (10^−6^ M) had no effect on it (Figure 3C).

### 2.2. Effects of Prostaglandin D_2_ (PGD_2_) on Cytosolic Ca^2+^ Level in Human Bronchial Smooth Muscle Cells (hBSMCs)

**Figure 4:**
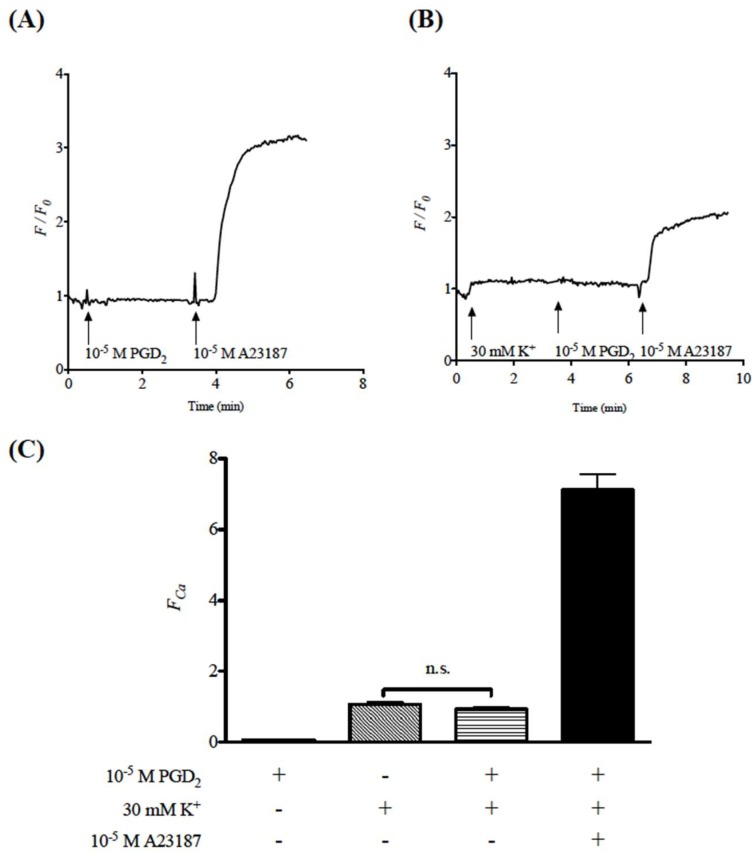
Effects of prostaglandin D_2_ (PGD_2_) on the cytosolic Ca^2+^ level in cultured human bronchial smooth muscle cells (hBSMCs) determined by a fluorescent Ca^2+^ indicator Fluo-8. (**A**,**B**) Representative trace of change in cytosolic Ca^2+^ (*F*/*F*_0_, ratio of the Ca^2+^ fluorescence intensity to that at time 0 (baseline)). The Fluo-8-loaded hBSMCs were stimulated with 30 mM K^+^ and, when its stable response was observed, 10^−5^ M PGD_2_ was applied. To confirm the maximal response, a Ca^2+^ ionophore A23187 (10^−5^ M) was applied at the end of experiments. (**C**) Summary of normalized ratios of the Ca^2+^ fluorescence intensities (*F*_Ca_) data. Results are presented as mean ± SEM from 8 independent experiments. Note that neither the baseline Ca^2+^ level nor the stable increase in Ca^2+^ induced by K^+^ depolarization was affected by PGD_2_.

Due to the difficulty in preparing isolated BSM cells with high purity from the mouse tissues, change in cytosolic Ca^2+^ level was measured using commercially available human BSM cells (hBSMCs) in the present study. The hBSMCs were loaded with a green fluorescent Ca^2+^ indicator, Fluo-8 [16]. As shown in Figure 4A,B, in the hBSMCs incubated with Fluo-8/AM, stimulation of the cells with a Ca^2+^ ionophore A23187 (10^−5^ M) caused a marked increase in *F*/*F_0_*, that is, an increase in cytosolic Ca^2+^ concentration, indicating a successful loading of Fluo-8 into the cells. In the Fluo-8-loaded hBSMCs, stimulation of the cells with 30 mM K^+^ caused a slight but distinct increase in cytosolic Ca^2+^ concentration (Figure 4B,C). Interestingly, PGD_2_ had no effect on the K^+^ depolarization-induced increase in cytosolic Ca^2+^ level (Figure 4B,C). PGD_2_ also did not alter the basal cytosolic Ca^2+^ level in the Fluo-8-loaded cells (Figure 4A).

### 2.3. Activation of RhoA/Rho-Kinase Signaling by Prostaglandin D_2_ (PGD_2_)

**Figure 5:**
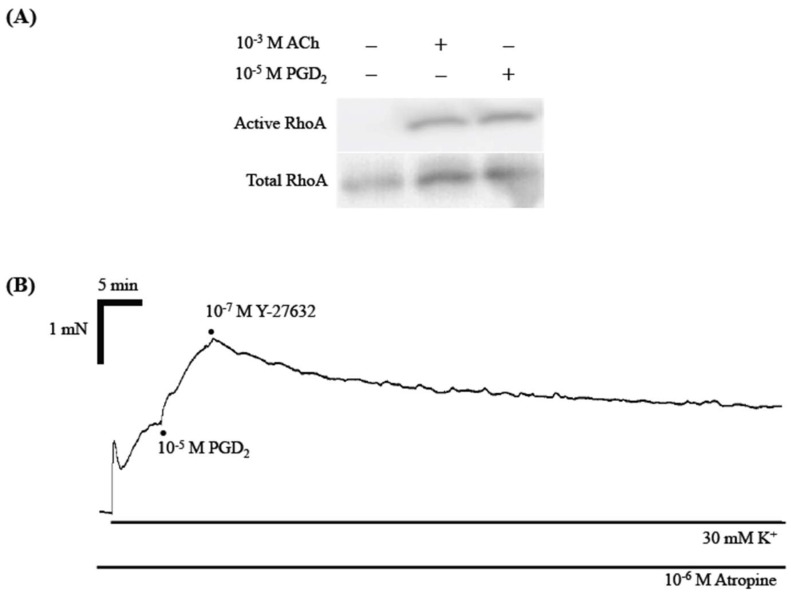
Activation of RhoA/Rho-kinase signaling by prostaglandin D_2_ (PGD_2_) in bronchial smooth muscle (BSM) of the mouse. (**A**) Effect of PGD_2_ on the level of GTP-bound active form of RhoA. Freshly isolated BSMs of mice were stimulated with acetylcholine (ACh: 10^−3^ M) or PGD_2_ (10^−5^ M) for 10 min, and GTP-RhoA pull-down assay and RhoA immunoblottings were performed as described in Materials and Methods section. The blots for GTP-bound (active: *upper*) and total RhoA (*lower*) are shown and representative from 3 independent experiments, respectively. (**B**) Effect of Y-27632 (a selective Rho-kinase inhibitor) on the augmented contraction induced by prostaglandin D_2_ (PGD_2_) in BSMs isolated from mice. After the BSM contraction induced by PGD_2_ reached to plateau, 10^−7^ M Y-27632 was applied. A trace of change in the active force is shown and representative from 3 independent experiments.

The results that PGD_2_ caused an augmentation of contraction (Figure 2B,C) under the constant cytosolic Ca^2+^ level (Figure 4) remind us of the Ca^2+^ sensitization of smooth muscle contraction. In smooth muscle cells including airways, activation of a monomeric G-protein, RhoA, causes Ca^2+^ sensitization of the contraction by activating its downstream Rho-kinases [17,18]. To determine whether PGD_2_ activates RhoA protein, the GTP-RhoA pull-down assay was performed in mouse BSMs stimulated by PGD_2_. As previously reported [19], acetylcholine (ACh: 10^−3^ M) stimulation caused an increase in GTP-bound, active form of RhoA protein in the BSMs of mice (Figure 5A). Similarly, as shown in Figure 5A, an increase in the active form of RhoA protein was observed when the BSM tissues were stimulated with 10^−5^ M PGD_2_, the concentration where no contractile response from baseline tone was observed (see above). The tension study also revealed an activation of RhoA/Rho-kinase signaling by PGD_2_: the PGD_2_-induced augmentation of contraction was blocked by Y-27632 (10^−7^ M), a selective inhibitor of Rho-kinases (Figure 5B).

## 3. Discussion

The current study was carried out to determine the role of prostaglandin D_2_ (PGD_2_) on smooth muscle function of the airways using the bronchial smooth muscles (BSMs) isolated from mice. Although PGD_2_ had no effect on their baseline tension, PGD_2_ significantly augmented the BSM contraction induced by high K^+^ depolarization (Figure 2B,C). The PGD_2_-induced augmentation of contraction was inhibited both by a DP_1_ antagonist, laropiprant, and a Rho-kinase inhibitor, Y-27632 (Figure 3 and Figure 5B). Furthermore, PGD_2_ could cause an activation of RhoA protein (Figure 5A). In the high K^+^-depolarized cultured human BSM cells, PGD_2_ caused no further increase in cytosolic Ca^2+^ concentration (Figure 4). These findings suggest that PGD_2_ acts on DP_1_ receptors to cause RhoA/Rho-kinase-mediated Ca^2+^ sensitization of contraction in BSMs.

PGD_2_ is an acidic lipid mediator derived from the metabolism of arachidonic acid by the action of cyclooxygenases and downstream PGD_2_ synthases, and is mainly released from mast cells when activated by antigen stimulation [20]. Allergen challenge to the airways caused an increase in PGD_2_ level in the airways of asthmatics [8,9]. However, the functional role of PGD_2_ on airway smooth muscle has not yet been unified. In tracheal smooth muscle strips isolated from the guinea pigs, PGD_2_ produced a concentration-dependent contraction [12]. Similarly, PGD_2_ caused a contraction in bronchial rings isolated from the dogs [13]. In contrast, PGD_2_, at a concentration of 3 µM, elicited a relaxation in the murine tracheal smooth muscle precontracted with carbachol [10]. Currently, PGD_2_ had no effect on basal tension in BSMs isolated from the mice (see Results section). Differences in the species, region (tracheal versus bronchial smooth muscles), and/or the experimental condition used may be involved in the difference in the PGD_2_ response in smooth muscles of the airways. Thus, note that the current study also contains a certain limitation: cultured human BSM cells (hBSMCs) were used for cytosolic Ca^2+^ measurement whereas functional studies were performed using mouse BSM tissues.

The current RT-PCR analyses showed expression of DP_1_ and DP_2_ receptors in BSM cells (Figure 1), indicating that PGD_2_ could directly act on BSM cells. Although PGD_2_ did not affect the basal tension, it augmented the submaximal contraction induced by 30 mM K^+^ in BSMs isolated from the mice (Figure 2). The augmented contraction induced by PGD_2_ was inhibited by laropiprant (Figure 3), a DP_1_ antagonist [14], but not by fevipiprant (see RESULTS), a DP_2_ antagonist [15]. An involvement of TP receptor in the PGD_2_-mediated contraction has also been suggested [21]. However, PGD_2_ did not increase cytosolic Ca^2+^ in the present study (Figure 4), whereas an induction of contraction with Ca^2+^ mobilization by the TP receptor activation has been demonstrated [22]. In addition, our preliminary study revealed that stimulation of TP receptors with a thromboxane A_2_ (TXA_2_) mimic, U46619, caused a distinct contraction from baseline tension (without K^+^ depolarization) in the mouse BSMs. Pretreatment of BSMs with ozagrel, an inhibitor of TXA_2_ synthase, also did not inhibit the augmented contraction induced by PGD_2_ (data not shown). It is thus unlikely that the TXA_2_/TP receptor is involved in the PGD_2_-mediated response in the mouse BSMs. Thus, an activation of DP_1_ receptors on the BSM cells might be responsible for the synergistic contraction induced by PGD_2_.

Currently, PGD_2_ augmented the contraction induced by high K^+^ depolarization in mouse BSM tissues (Figure 2B,C). In the high K^+^-depolarized cultured hBSMCs, PGD_2_ caused no further increase in cytosolic Ca^2+^ concentration (Figure 4). Collectively, these findings suggest that PGD_2_ augmented the BSM contraction induced by K^+^ depolarization without any increase in cytosolic Ca^2+^ concentration. The observation that PGD_2_ caused an augmentation of contraction under the constant cytosolic Ca^2+^ level reminds us of the Ca^2+^ sensitization of smooth muscle contraction. Indeed, the augmented contraction induced by PGD_2_ was inhibited by a Rho-kinase inhibitor, Y-27632 (Figure 5). In addition, stimulation of the BSMs with PGD_2_ caused an increase in the active form of RhoA, GTP-bound RhoA (Figure 5). The current study for the first time, to our knowledge, demonstrated that PGD_2_ activates the RhoA/Rho-kinase signaling to induce Ca^2+^ sensitization of contraction in the BSMs. Previous studies, including ours, demonstrated that muscarinic receptor stimulation of airway smooth muscle caused both an increase in cytosolic Ca^2+^ concentration and an activation of RhoA/Rho-kinase signaling, resulting in the contraction [17,23,24]. On the other hand, the current study revealed that PGD_2_ did not have the ability to increase cytosolic Ca^2+^ level in the BSMs (Figure 4). This may be a reason that PGD_2_ did not cause any contraction from the baseline tension: the cytosolic Ca^2+^ level at the baseline tension might not have been enough to induce BSM contraction even if the RhoA/Rho-kinase signaling was activated.

It is a remarkable event that the PGD_2_-induced augmentation of contraction was inhibited by laropiprant, an antagonist of DP_1_ receptor that is known as a Gs protein-coupled receptor. In smooth muscle cells including the airways, the Gs protein activation, such as beta-adrenoceptor stimulation by isoprenaline, causes an increase in cAMP level to induce relaxation [25,26,27]. However, the current study indicated that activation of DP_1_ receptor by PGD_2_ could cause a response to contractile direction. Although the discrepancy is not explainable now, an activation of extracellular signal-regulated kinase (ERK) signaling by DP_1_ receptor stimulation has also been reported in nasal epithelial cells [28]. It is thus possible that, in addition to the classical Gs/cAMP pathway, the DP_1_ receptor stimulation activates multiple intracellular signaling, including the RhoA/Rho-kinase signaling. Further studies are needed to make clear the mechanism of action of PGD_2_ in the BSMs.

In conclusion, the current study revealed that PGD_2_ augmented the BSM contraction by activating the RhoA/Rho-kinase-mediated Ca^2+^ sensitization of contraction via an activation of DP_1_ receptors on the BSM cells. Increased PGD_2_ level in the airways might be one of the causes of the enhanced airway responsiveness to nonspecific stimuli, one of the characteristic features of bronchial asthma.

## 4. Materials and Methods

### 4.1. Animals

Male BALB/c mice were purchased from the Tokyo Laboratory Animals Science Co., Ltd. (Tokyo, Japan) and housed in a pathogen-free facility. All animal experiments were approved by the Animal Care Committee of the Hoshi University, Tokyo, Japan (permission code: 30-086, permission date: 21 June 2018).

### 4.2. Pharmacological Reagents

Prostaglandin D_2_ (PGD_2_: Cat. No. 12010) and laropiprant (MK-0524: Cat. No. 10009835) were purchased from Cayman Chemical (Ann Arbor, MI, USA). Fevipiprant was purchased from MedChem Express (Monmouth Junction, NJ, USA: Cat. No. HY-16768).

### 4.3. Determination of Bronchial Smooth Muscle (BSM) Responsiveness

Mice were sacrificed by exsanguination from abdominal aorta under urethane (1.6 g/kg, *i.p.*) anesthesia and the airway tissues under the larynx to lungs were immediately removed. About 3 mm length of the left main bronchus (about 0.5 mm diameter) was isolated. The resultant tissue ring preparation was then suspended in a 5 mL organ bath by two stainless-steel wires (0.2 mm diameter) passed through the lumen. For all tissues, one end was fixed to the bottom of the organ bath while the other was connected to a force-displacement transducer (TB-612T, Nihon Kohden, Tokyo, Japan) for the measurement of isometric force. A resting tension of 0.5 g was applied. The buffer solution contained modified Krebs–Henseleit solution with the following composition (mM): NaCl 118.0, KCl 4.7, CaCl_2_ 2.5, MgSO_4_ 1.2, NaHCO_3_ 25.0, KH_2_PO_4_ 1.2, and glucose 10.0. The buffer solution was maintained at 37 °C and oxygenated with 95% O_2_/5% CO_2_. After the equilibration period, the tension studies were performed. In case of the high K^+^ depolarization studies, experiments were conducted in the presence of atropine (10^−6^ M).

### 4.4. Determination of Active Form of RhoA in BSM

The active form of RhoA, GTP-bound RhoA, in BSMs was measured by GTP-RhoA pull-down assay as described previously [19]. In brief, the isolated main bronchial tissues were equilibrated in oxygenated Krebs–Henseleit solution at 37 °C for 1 h. After the equilibration period, the tissues were stimulated with PGD_2_ (10^−5^ M) or ACh (10^−3^ M) for 15 min, and were quickly frozen with liquid nitrogen. The tissues were then lysed in lysis buffer with the following composition (mM): HEPES 25.0 (pH 7.5), NaCl 150, IGEPAL CA-630 1%, MgCl_2_ 10.0, EDTA 1.0, glycerol 10%, 1× protease inhibitor cocktail (Nakalai tesque, Kyoto, Japan), and 1× phosphatease inhibitor cocktail (Nakalai tesque). Active RhoA in tissue lysates (200 µg protein) was precipitated with 25 µg GST-tagged Rho binding domain (amino acids residues 7–89 of mouse rhotekin; Upstate, Lake Placid, NY, USA), which was expressed in *Escherichia coli* and bound to glutathione-agarose beads. The precipitates were washed three times in lysis buffer, and after adding the SDS loading buffer and boiling for 5 min, the bound proteins were resolved in 15% polyacrylamide gels, transferred to nitrocellulose membranes, and immunoblotted with rabbit polyclonal anti-RhoA (Abcam, Cambridge, UK) as primary antibodies.

### 4.5. Cell Culture and [Ca^2+^]_cyto_ Measurement

Normal human BSM cells (hBSMCs; a male donor: purchased from Cambrex Bio Science Walkersville, Inc., Walkersville, MD, USA) were maintained in SmBM medium (Cambrex Bio Science Walkersville, Inc., Walkersville, MD, USA) supplemented with 5% fetal bovine serum, 0.5 ng/mL human epidermal growth factor (hEGF), 5 µg/mL insulin, 2 ng/mL human fibroblast growth factor-basic (hFGF-b), 50 µg/mL gentamicin, and 50 ng/mL amphotericin B. Cells were maintained at 37 °C in a humidified atmosphere (5% CO_2_), fed every 48–72 h, and passaged when cells reached 90–95% confluence. Then the hBSMCs (passages 5–7) were seeded in 24-well plates (Becton Dickinson Labware, Franklin Lakes, NJ, USA) and were cultured without serum. Twenty-four hours after the starvation period, the cells were loaded with Fluo-8/AM (2.5 M: AAT Bioquest, Inc., Sunnyvale, CA, USA) in serum-free SmBM medium for 90 min at 37 °C. The cells were washed with PBS and maintained in Krebs–Henseleit solution described above. The intracellular Fluo-8 fluorescence was monitored using fluorescence microscope (Keyence, Osaka, Japan) with BZ-X filter GFP (470/40, 535/50 nm). Images were pictured using time-lapse imaging (Keyence), and analyzed with BZ-X analyzer (Keyence). Change in the cytosolic Ca^2+^ level was calculated as ratio to the basal fluorescence intensity.

### 4.6. RT-PCR Analyses

Total RNAs of hBSMCs and mouse BSM tissues were extracted using NucleoSpin™ miRNA (TaKaRa Bio, Inc., Shiga, Japan) according to the manufacturer’s instruction. cDNAs were prepared from the total RNA by using PrimeScript™ RT reagent Kit (TaKaRa) according to the manufacturer’s instructions. cDNA samples were subjected to PCR with Quick Taq™ HS DyeMix (TOYOBO Co., Ltd., Osaka, Japan) in a final volume of 10 µL. The PCR primer sets used are shown in Table 1 (for human) and Table 2 (for mouse), which was designed from published database, BLAST. The thermal cycle profile used was (1) denaturing for 30 s at 94 °C, (2) annealing primers for 30 s at 60 °C, (3) extending the primers for 1 min at 68 °C, and the reaction was run for 40 cycles. The PCR products were subjected to electrophoresis on 2% agarose gel and visualized by ethidium bromide staining.

### 4.7. Statistical Analyses

All the data are expressed as means ± SE. Statistical significance of difference was determined by paired *t*-test (Figure 2B,C) or one-way analysis of variance (ANOVA) with post hoc Bonferroni’s multiple comparison (Figure 3B and Figure 4C) using Prism 5 for Mac OS X (GraphPad Software, La Jolla, CA, USA). A value of *p* < 0.05 was considered significant.

## Figures and Tables

**Table 1:** Primer sequences for RT-PCR used in the present study (human).

Gene Name	RefSeq Accession		Sequence	Amplicon Size
human *PTGDR*	NM_000953	Sense	5′-TCTGCGCGCTACCTTTCATG-3′	85 bp
		Antisense	5′-TCCTCGTGGACCATCTGGATA-3′	
human *PTGDR2*	NM_004778	Sense	5′-CCTCTGTGCCCAGAGCCCCACGATGTCGGC-3′	114 bp
		Antisense	5′-ATGTAGCGGATGCTGGTGTTG-3′	
human *PTGER1*	NM_000955	Sense	5′-GATGGTGGGCCAGCTTGTC-3′	72 bp
		Antisense	5′-GCCACCAACACCAGCATTG-3′	
human *PTGER2*	NM_000956	Sense	5′-GTGCTGACAAGGCACTTCATGT-3′	87 bp
		Antisense	5′-TGTTCCTCCAAAGGCCAAGTAC-3′	
human *PTGER3*	NM_198714	Sense	5′-AAGGCCACGGCATCTCAGT-3′	76 bp
		Antisense	5′-TGATCCCCATAAGCTGAATGG-3′	
human *PTGER4*	NM_000958	Sense	5′-CTTGGAGGCAGGAATTTGCTT-3′	77 bp
		Antisense	5′-AAAGTCCTCAGTGAGGTGGTGTCT-3′	
human *PTGFR*	NM_000959	Sense	5′-GCACATTGATGGGCAACTAGAA-3′	91 bp
		Antisense	5′-GCACCTATCATTGGCATGTAGCT-3′	
human *PTGIR*	NM_000960	Sense	5′-GCCGATCAGCTGCTGTTTCT-3′	75 bp
		Antisense	5′-TTTCCTCTGTCCCTCACTCTCTTC-3′	
human *TBXA2R*	NM_001060	Sense	5′-ACGGAGAAGGAGCTGCTCATC-3′	84 bp
		Antisense	5′-GCGGCGGAACAGGATATACA-3′	
human *GAPDH*	NM_002046	Sense	5′-GGAGCCAAAAGGGTCATCATCTC-3′	282 bp
		Antisense	5′-AGGGATGATGTTCTGGAGAGCC-3′	

**Table 2:** Primer sequences for RT-PCR used in the present study (mouse).

Gene Name	RefSeq Accession		Sequence	Amplicon Size
mouse *Ptgdr1*	NM_008962	Sense	5′-CAACCTGGGTGCCATGTAC-3′	112 bp
		Antisense	5′-GGACCCGTGCCTGTAGTCT-3′	
mouse *Ptgdr2*	NM_009962	Sense	5′-CTGCACCTGGCGCTATC-3′	174 bp
		Antisense	5′-GTCCAGGCTAATGGCACT-3′	
mouse *Ptger1*	NM_013641	Sense	5′-TACATGGGATGCTCGAAACA-3′	223 bp
		Antisense	5′-TTTTAGGCCGTGTGGGTAG-3′	
mouse *Ptger2*	NM_008964	Sense	5′-ATGCACCTGCTGCTTATCGT-3′	196 bp
		Antisense	5′-TAATGGCCAGGAGAATGAGG-3′	
mouse *Ptger3*	NM_001359745	Sense	5′-TGCTGGCTCTGGTGGTGAC-3′	258 bp
		Antisense	5′-ACTCCTTCTCCTTTCCCATCTGTG-3′	
mouse *Ptger4*	NM_001136079	Sense	5′-CCATCGCCACATACATGAAG-3′	209 bp
		Antisense	5′-TGCACAGATGGCGAAGAGTG-3′	
mouse *Ptgfr*	NM_008966	Sense	5′-CTGCTCCGGACACAACCACTC-3′	191 bp
		Antisense	5′-GGTTCTCCGTCTGGCAGGTTG-3′	
mouse *Ptgir*	NM_008967	Sense	5′-GGATGAAGTTTACCACCTGATTCTGC-3′	196 bp
		Antisense	5′-AGCCTTTCGGAAAAGGATGAAGAC-3′	
mouse *Tbxa2r*	NM_009325	Sense	5′-TTTCGCCCGGTGAACATC-3′	255 bp
		Antisense	5′-GGCTCGCCAGTCCAACAA-3′	
mouse *Gapdh*	NM_001289726	Sense	5′-CCTCGTCCCGTAGACAAAATG-3′	100 bp
		Antisense	5′-TCTCCACTTTGCCACTGCAA-3′	

## Abbreviations

ACh	acetylcholine
AHR	airway hyperresponsiveness
ANOVA	analysis of variance
BAL	bronchoalveolar lavage
BSM	bronchial smooth muscle
COX	cyclooxygenase
CRTH2	chemoattractant receptor-homologous molecule on Th2 cells
ERK	extracellular signal-regulated kinase
*F*_Ca_	normalized ratios of the Ca^2+^ fluorescence intensities
*F*/*F*_0_	ratio of the Ca^2+^ fluorescence intensity to that at time 0 (baseline)
Fluo-8/AM	Fluo-8 acetoxymethyl ester
GPCR	G protein-coupled receptor
GST	glutathione S-transferase
GTP	guanosine triphosphate
PG	prostaglandin
SDS	sodium dodecyl sulfate
TXA_2_	thromboxane A_2_
